# Reducing emergency department length of stay for hematology patients of tikur anbessa specialized hospital: An improvement initiative

**DOI:** 10.1371/journal.pone.0329316

**Published:** 2025-09-08

**Authors:** Merahi Kefyalew Merahi, Rahel Argaw, Aschalew Worku, Tsegaw Molla, Biruk Abayneh, Natnael Mathewos, Be’emnet Amare, Selamawit Kassahun, Kefelegn Negalign Mekuria, Matyas Wondwossen Elssa

**Affiliations:** Addis Ababa University, College of Health Science, Addis Ababa, Ethiopia; Kuwait Ministry of Health, KUWAIT

## Abstract

**Introduction:**

Prolonged Emergency Department (ED) stays, a global issue driving overcrowding, were exacerbated at our hospital by lab delays and extended waits, increasing patient stress. This study aimed to reduce hematology patients’ length of stay (LOS). Using the fishbone method to identify care barriers, three interventions were implemented: redesigned lab referral systems, an online specialist communication platform, and patient navigation floor maps.

**Methods:**

At Tikur Anbessa Specialized Hospital (Ethiopia), a quality improvement initiative targeted hematology patients (n = 203 baseline; n = 63 post-intervention) with prolonged emergency department (ED) stays. Using two PDSA cycles, interventions included an online consultation platform, floor markings for navigation, and digitizing peripheral smear workflows via the I-Care system. Weekly data on consultation time, lab turnaround time (TAT), navigation errors, and length of stay (LOS) were analyzed with run charts and Interrupted Time Series (ITS) regression.

**Results:**

Median LOS decreased by 62.5% (144–54 hours; p < 0.001), remaining stable during a 5-week pause. Consultation time fell 80% (12 to 2.4 hours; 95% CI: 1.8–3.0), and lab TAT improved by 70% (78 to 23.25 hours). Navigation errors dropped from 53% to ≤7%, with minor fluctuations. Clinical outcomes (e.g., mortality) were not assessed, and long-term sustainability requires further study.

**Conclusions:**

Targeted interventions improved care and efficiency at Tikur Anbessa Hospital, but sustained reductions in ED LOS were limited by data gaps and discontinued initiatives. Future efforts in resource-limited settings should prioritize continuous monitoring, stakeholder collaboration, and staff well-being.

## Introduction

Emergency department (ED) length of stay (LOS) is defined as the time from when a patient is registered to the time the patient is either admitted to a hospital bed or discharged from the ED [1]. According to the Ethiopian hospitals guideline, patients requiring emergency services should be kept at the ED for a maximum of 24 hours. Any patient who needs care for longer than 24 hours should be transferred to a ward for inpatient management [2,3]. Recent research indicates that Ethiopian hospital emergency departments are currently overcrowded with patients, putting them at risk of severe problems [4,5]. This overcrowding is driven by several factors, including lack of insurance, bed availability, acuity level, presentation without communication, delayed consultation, shift change experience, and the need for admission, all of which contribute to increased ED LOS [6,7].

Emergency departments (EDs) globally are faced with increasing patient loads and expectations, due to the annual ED visits rapidly increasing with the growing population, which affects emergency care as well as the length of stay [8,9]. Previous studies have reported prolonged ED LOS rates of 4% in England, 72.5% in Botswana, and 91.5% in Southern Ethiopia, with the highest rates observed in Lower-Income Countries [10–12].

Studies have shown that prolonged stay in the emergency department can adversely affect patient outcomes, leading to increased length of hospital admission, higher mortality, and higher inpatient costs [1,5,6,13]. The mortality rate increases even further in patients who are boarded in the emergency for more than 24 hours, and those who are elderly [1].

We aim to decrease the length of stay for patients in the ED by systematically working on each department. For this study, we focus on hematology patients to illustrate the impact of our interventions in that specific group. The project focuses on mitigating navigational challenges within the hospital’s complex infrastructure, which currently hampers timely patient placement and care. By improving patient navigation and resource management, we seek to enhance the efficiency of the ED. To systematically implement these improvements, we will employ the Plan-Do-Study-Act (PDSA) methodology. This iterative approach allows for continuous testing and refinement of interventions to achieve our objectives.

### Problem

Recent research indicates that prolonged stay of adult patients at emergency department was found to be high based on Ethiopian target emergency department patient length of stay [6]. Prolonged ED stay increases mortality by 15–30% and admitted patients held in the ED longer may die more frequently than those admitted quickly and patients will leave without being seen [14,15]. Longer ED length of stay (LOS) may compromise the quality of care and delay the emergency evaluation of other patients [16,17]. Long ED stay will also increase health care cost on patients or health care systems [18], inpatient length of stay, and the risk of nosocomial infections [16,19].

The ED of our hospital has been experiencing several challenges that affect patient care and satisfaction. These challenges include long waiting times, patient confusion, and delays in laboratory procedures. Patients often struggle to navigate the ED, leading to unnecessary stress and frustration. The turn-around time for laboratory procedures, such as the peripheral smear, has also been a concern, leading to delays in diagnosis and treatment. These problems have highlighted the need for innovative solutions to improve patient care and satisfaction in the ED.

### Aim

The team aimed to decrease the length of patient stay from 144 Hrs to 56 Hrs from September 1, 2022 to February 29, 2023 by focusing on three objectives: improving the time to consultation, reducing the turn-around time for peripheral smear, and decreasing the time needed for navigating offices. To monitor progress and ensure success, the team initiated and closely monitored three quality improvement projects.

## Methods

### Context

Tikur Anbessa Specialized Hospital is a public hospital in Addis Ababa, Ethiopia. The hospital is one of the largest and oldest hospitals in the country and serves as a referral center for patients from across Ethiopia. The hospital was built with funds from the entire Ethiopian people and has been providing services for all community. It has now reached treating over 500 thousand outpatients and more than 21 thousand inpatients annually [20]. The emergency department at Tikur Anbessa Specialized Hospital is one of the busiest emergency departments in Ethiopia, treating 900–1000 patients every month. Among these, nearly 100 are hematology patients. A hematology patient is someone receiving medical care for blood-related disorders, such as anemia, leukemia, clotting issues, or bleeding disorders. The department has 10 attending physicians, 12 residents, 24 interns and 55 nurses to care for many patients, including those with critical and life-threatening conditions.

The emergency department at Tikur Anbessa Hospital is divided into different sections, including triage, observation, and treatment. The triage section is the first point of contact for patients who come to the emergency department. Here, patients are assessed and prioritized based on the severity of their condition. The observation section is where patients who do not require immediate medical intervention are monitored for a period of time before being discharged or transferred to another department for further treatment. The treatment section is where patients receive medical care for various conditions, including trauma, infections, and chronic diseases.

The number of doctors working in the emergency department at any given time depends on several factors, including the time of day, the day of the week, and the patient volume. During peak hours, such as daytime and evening hours, the emergency department is usually staffed by several attending physicians, resident physicians, and interns. The exact number of doctors on duty may vary. Still, at least one or two attending physicians, four or five residents, and a few interns are typically working in the department. During off-peak hours, such as during the overnight hours, the number of doctors on duty may be reduced, with one attending physician and a few residents and interns available to care for patients.

### Study design

We conducted a quality improvement study at Tikur Anbessa Specialized Hospital (TASH) aimed at reducing the length of stay for hematology patients in the emergency department (ED). The study employed a mixed-methods approach, incorporating both quantitative and qualitative data collection.

### Baseline assessment

To establish a baseline length of stay, we performed a retrospective analysis of hematology patients who visited the ED from May 16/2022 to July 13/2022. A total of 203 hematology patients were seen during this time. The Shapiro-Wilk test revealed a significant deviation from normality (p < 0.05), leading to the use of the median as the measure of central tendency.

### Assessment of the problem

The project team aimed to assess the problem by following hematology patients from the time they entered the hospital until they were discharged to identify the factors that contribute to long waiting times and develop solutions to improve the patient experience. In order to achieve that 10 individuals were assigned from the team to collect and document the data weekly. All hematology patients, a total of 63, who visited the ED during the study time from July 15/2022 to July 29/2022 were included. The project team used a mixed-methods approach to assess the problem. This involved both quantitative and qualitative data collection methods.

The methods used were mainly observations. The project team followed patients from the time they entered the hospital until they were discharged, sent to the ward for more care or, in some cases, left against medical advice. We collected data on the following: patient demographics, including age, gender, and reason for visit; waiting times, such as the time when patients arrived at the hospital, the time when they were seen by a medical professional, the time when patients were sent to laboratory and imaging tests, the time when drugs were administered, and the time when patients left the emergency department; and patient satisfaction, which was collected through surveys on waiting time, quality of care, and overall patient experience.

**Fig 1:**
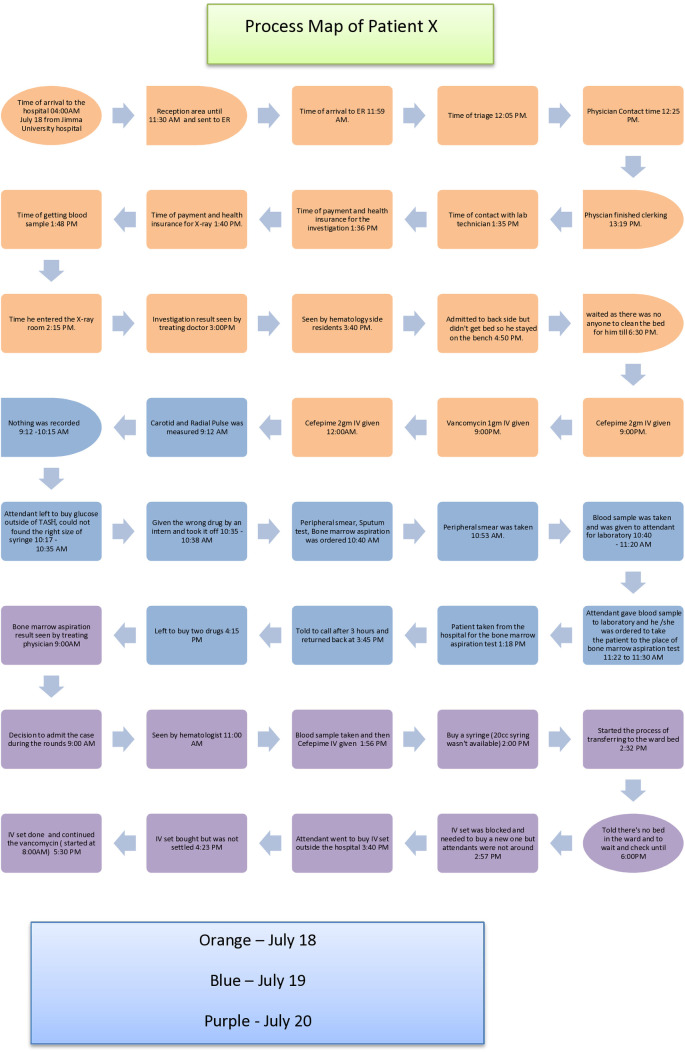
Process map of a hematology patient in the ED.

**Fig 2:**
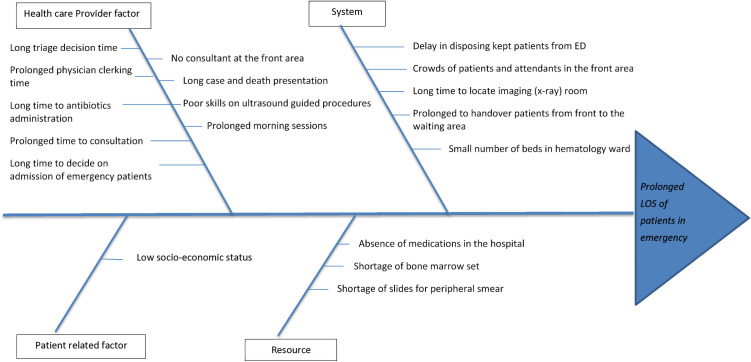
Fishbone diagram representation of the problems identified from the process maps.

The process map outlining the steps a hematology patient undergoes when attending the emergency department at Tikur Anbessa Hospital is as follows (Fig 1):

From the process map, the project team listed all the factors that fell below the quality of care standard. Then, we classified the different problems in the hospital based on general factors using the fishbone method (Fig 2).

### Ethics approval and consent to participate

This quality improvement study, conducted at the Emergency Department of Tikur Anbessa Specialized Hospital, was reviewed and ethically approved by the Clinical Governance and Quality Improvement Directorate of Tikur Anbessa Specialized Hospital, as it qualifies as a quality improvement initiative rather than a research study. Informed consent was waived, as all interventions fell within the scope of routine clinical care, with no direct patient interaction or collection of personally identifiable information. Only process and outcome metrics were analyzed, and all data were anonymized. The study complies with institutional ethical standards and the Declaration of Helsinki.

### Interventions

**Fig 3:**
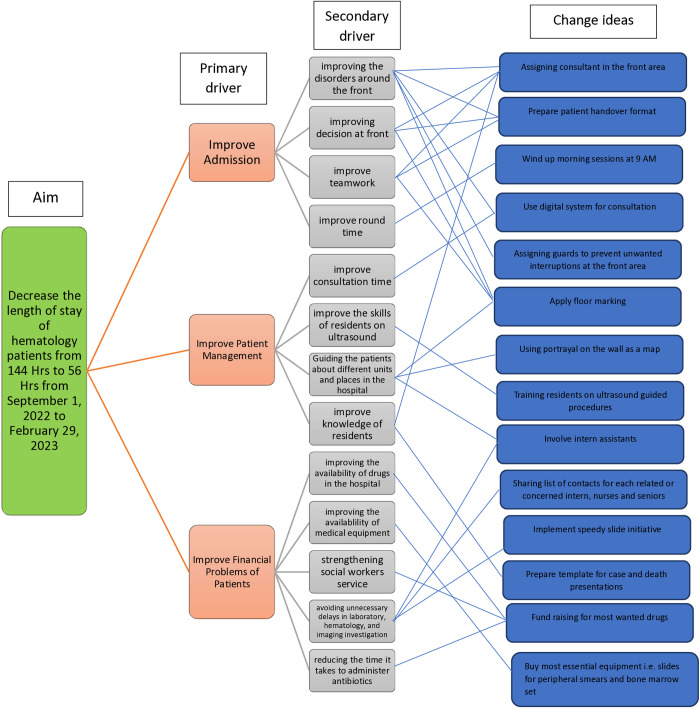
Driver diagram for strategies to reduce Length of stay of hematology patients in the ED.

The project team developed a series of change ideas to address the identified problems and gaps in the service provision of the emergency department. These change ideas included redesigning the system of sending patients to the hospital’s lab for peripheral morphology tests, implementing a floor map to improve patient navigation, and implementing an online platform for communication with senior specialists, etc. We developed driver diagrams to brainstorm and summarize the change ideas (Fig 3).

Following this, we developed a questionnaire that included the list of problems derived from the fish bone diagram. We then asked two consultants, five residents, six nurses, and four interns to prioritize these issues by ranking each one based on three criteria: the severity of the issue, the importance and potential impact of addressing it, and the feasibility of implementing solutions. Respondents used a scale of one to five for each parameter. The project team then prioritized the change ideas with the highest average total score calculated from the ratings provided by the respondents taking into consideration the resources these interventions require (Table 1).

**Table 1:** Problem prioritization questionnaire rankings.

S/N	Problem List	Criteria for Rank			Total Score	Rank
		Frequency/ Magnitude	Importance	Feasibility		
1	Long triage decision time	3.00	3.33	3.50	10.83	5
2	Prolonged physician clerking time	2.83	3.17	3.5	9.50	11
3	Prolonged time to antibiotics	3.17	3.83	3.00	10.00	8
4	Prolonged time to consultation	3.77	3.90	3.50	11.17	1
5	No senior physician in the front area	3.33	3.75	2.92	10.00	8
6	Long case and death presentation	2.50	2.67	3.17	7.17	14
7	Poor skill in ultrasound-guided procedures	2.50	2.67	3.17	8.33	13
8	Prolonged morning sessions	3.17	3.50	3.33	10.25	7
9	Crowded front area with attendants and patients on clerk time	3.50	3.50	3.00	10.50	6
10	Low socioeconomic status of patients	3.65	3.48	2.37	9.50	11
11	Absence and shortage of highly essential equipment- Bone marrow set and slide for peripheral smear	4.13	3.94	3.10	11.17	1
12	Small number of beds in hematology ward	4.00	4.00	3.00	11.00	4
13	Absence of medications in the hospital	3.67	3.33	2.67	9.67	10
14	Long time to locate X- ray room	3.64	3.86	3.56	11.06	3
15	Long time to decide on admission of emergency patients	2.00	2.33	3.64	6.97	16
16	Delay in disposing kept patients from ED	1.83	2.17	3.54	6.54	17
17	Prolonged to handover patients from front to the waiting area	2.17	2.50	3.33	7.06	15

The team selected a few key change ideas to implement initially, with plans to evaluate the effect and feasibility before implementing additional changes.

The first intervention improved the process for ordering and processing peripheral morphology tests. Previously, physicians could not order smears electronically, and patients had to leave the hospital to purchase slides, causing delays. Now, physicians can order smears directly through I-care, the electronic medical record (EMR) system, and the hospital provides the slides. The laboratory technician uses a CBC sample to prepare the smear, enabling hematologists to review results faster. This intervention was chosen to create a more efficient process and reduce the turn-around time for patient results.

**Fig 4:**
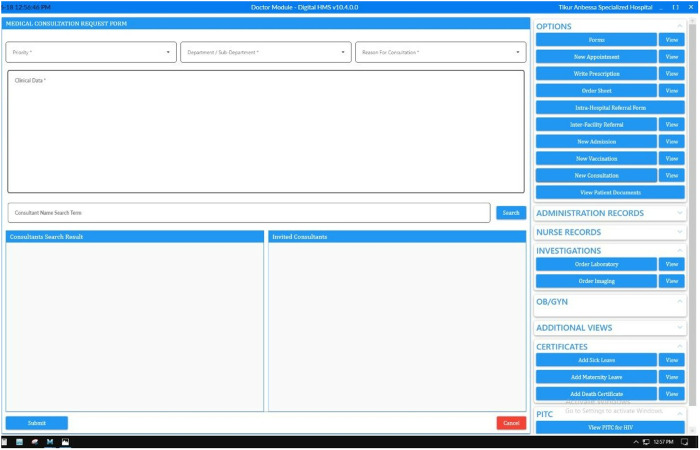
The newly designed online consultation platform.

The second intervention aimed to implement an online platform for communication with senior specialists to improve the consultation platform by allowing for more timely and efficient communication to reduce the time for consultation (Fig 4).

**Fig 5:**
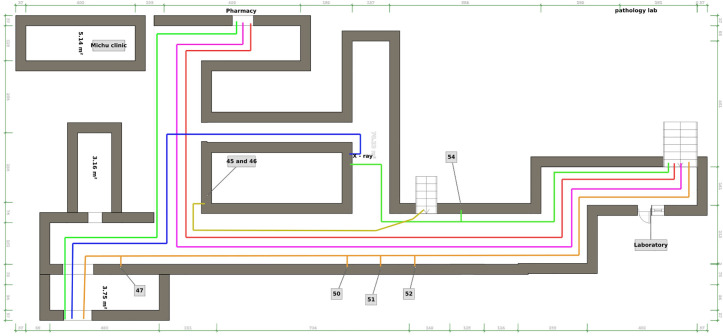
Floor map showing the way from the ED to the X-ray room.

In addition, we implemented a floor map to improve patient navigation (Fig 5). A floor map was developed to guide patients from the Front area (where patients are sent for investigation) to the X-ray room. This helped to reduce confusion and improve patient navigation in the department.

The rationale for choosing the three interventions over the others was based on several factors. First, the interventions were selected because they addressed the highest-ranked problems in the priority-setting table. By addressing these problems, there is a greater likelihood that the interventions will significantly reduce the length of stay of patients in the emergency department. Second, the interventions were chosen because they were considered the most cost-effective and non-radical changes. This means they were relatively easy to implement without causing significant disruption to the existing processes and incurring substantial costs. This made them more feasible for implementation and, therefore, more likely to be successful.

Third, the brainstormed alternative solutions, such as enforcing the hematology specialists to always be on call or reviewing slides more quickly, were deemed too vigorous and could potentially affect several concerned parties. These solutions could have posed significant obstacles to their implementation and may have resulted in less overall impact on the improvement of patients’ length of stay. Lastly, the Speedy Slides initiative was chosen over several proposed solutions due to its efficiency and more significant expected impact over the others. This initiative leverages the abundance of lab slides in the emergency laboratory and reduces the workload of the hematology department staff to prepare them. This is expected to result in faster test results, which can lead to quicker diagnosis and treatment, further reducing patients’ length of stay in the emergency department.

**Identifying process and outcome measures:** The project team identified the process measures for each intervention. The turn-around time of the patient’s results was identified as the process measure for the speedy slides initiative. The time for consultation was identified as the process measure for the online consultation platform intervention. The number of patients who made a wrong turn on their way to the X-ray room was used as a process measure for the floor mapping intervention. The outcome measure for the project was the length of stay of patients in the emergency department.

**Baseline measurement for the process measures:** A cross-sectional study was conducted from July 31 to August 7, 2023, including all 32 hematology patients visiting the emergency department during this period. Data on consultation time was collected by trained interns to ensure consistency. Among these patients, 15 required X-rays and were included in the analysis of navigational errors, recorded through direct observation and analyzed as weekly proportions (patients making wrong turns vs. total observed). Baseline turnaround time (TAT) for peripheral smears was established the week prior to initiating PDSA Cycle 2 (July 24–30, 2023) through retrospective review of laboratory records.

**Design:** The project team selected the PDSA cycle design as the design for this project. This design allowed for iterative, continuous improvement by testing and implementing changes and evaluating their impact before making further changes. The PDSA cycle is a flexible and adaptable design used to test and implement changes in various healthcare settings. It is particularly useful for quality improvement projects to address complex problems or improve care processes. Each PDSA cycle lasted one week, and the project was conducted over 24 weeks.

### Strategy

#### PDSA cycle 1.

During the first PDSA cycle, we implemented an online consultation platform and floor markings simultaneously. The method of evaluation that was in action for the application of the consultation platform comprised of the listed factors below:

**Data collection:** The study involved weekly measurement of the time to consultation, assessed by the length of stay in the hematology department. The data was obtained by analyzing the time gap from when the patient was linked to the hematology department in the I-Care system up to when the patient was seen in the hematology department.

**Data analysis:** For PDSA Cycle 1, the weekly collected data on length of stay (LOS), time to consultation, and the percentage of patients making wrong turns on their way to the X-ray room were analyzed to make amendments for the coming weeks. The median was used as the measure of central tendency for LOS and time to consultation data since they did not follow a normal distribution when assessed using the Shapiro-Wilk test in SPSS. These values were then followed with run charts to identify shifts and trends over time.

**Process measure:** The time to consultation was the process measure of the study, which was used to assess the effectiveness of the consultation platform. The weekly measurement of the length of stay was analyzed to determine whether the redesigned consultation platform reduced the time to consultation.

**Outcome measure:** The length of stay for hematology patients in the emergency department, which served as the outcome measure, was recorded weekly by the assigned interns in the department.

**Stakeholder feedback:** Feedback from emergency staff and on-call hematology department specialists was collected regularly to assess their satisfaction with the consultation platform and identify any areas for improvement. However, some drawbacks occurred at several data points of the PDSA cycle due to some hematology department specialists not responding through the newly designed I-Care platform. This caused the process measure to increase for that week. Additional meetings with the personnel were conducted to alleviate the issues, and advocacy was done to encourage the use of the platform. Overall, stakeholder feedback helped identify areas for improvement and allowed for the necessary adjustments to be made to the consultation platform.

The second intervention that was implemented during PDSA 1 was the application of floor marking from the emergency to the X-ray. This was intended to guide patients in the right direction and avoid unnecessary struggle and wasting of energy to find designated offices. Initially, these markings were created using tape stickers, and directional signs were drawn on the walls. However, starting in the 13th week, the stickers were replaced with floor paint, as the stickers tended to detach easily, creating confusion for patients.

The planned intervention was analyzed weekly according to the PDSA cycle, and along with it, the process and balancing measures were constantly recorded. The weekly process measure provided enough trends on the control chart to make possible changes in the coming weeks to keep the created change going. The truthfulness of the data was confirmed by asking the volunteers additional questions about the data they had collected.

The process measure and balancing measure were recorded each week to determine any changes in the efficiency of the intervention and the data analysis process. The data collected was analyzed using control charts to observe any trends or patterns in the effectiveness of the floor marking in reducing patient confusion and improving patient navigation. Possible implications to the data were identified, and viable solutions for the next cycle were outlined weekly to improve the process measure of the new system.

#### PDSA cycle 2.

The method of evaluation that was in action for the speedy slides initiative comprised of the listed factors below:

**Data collection:** The outcome measure was the length of stay of hematology patients in the emergency department, measured each week by assigned interns until the digitalized recording system was put to work. The process measure, the turn-around time to peripheral smear, was analyzed weekly from the digitalized platform that the laboratory technicians continuously record. The data was collected by an emergency senior specialist who led the project. The data collected was recorded accurately and consistently each week.

**Data analysis:** The same approach as in PDSA Cycle 1 was used for analyzing time to consultation and wrong turns, with weekly median calculations. Initially, the turnaround time for peripheral smears was planned to be monitored using run charts. However, due to the intervention being discontinued two weeks after its initiation and remaining inactive for five weeks, we adjusted our approach. Instead of using run charts, we compared the baseline median to the six-week median (excluding the five-week discontinuation period) to assess the intervention’s impact. Additionally, given the five-week interruption of the speedy slide initiative between Weeks 16 and 20, Interrupted Time Series (ITS) analysis was employed to more accurately assess the intervention’s impact. ITS was specifically chosen because it accounts for interruptions, enabling the evaluation of trends before, during, and after the disruption, and thus providing a clearer picture of the intervention’s effectiveness.

**Process measure:** The turn-around time to peripheral smear was the process measure of the study, which was used to assess the new system’s effectiveness. Data was collected on the time it took from when a peripheral smear was ordered for a patient to the time the result arrived, in order to establish the baseline turnaround time (TAT), one week prior to the initiation of PDSA 2. After establishing the baseline, the turnaround time was analyzed weekly to determine whether the newly designed system reduced the length of stay for hematology patients in the emergency department.

**Outcome measure:** The outcome measure, which was the length of stay of hematology patients in the emergency department, was measured each week by the assigned interns at the emergency department.

**Stakeholder feedback:** Feedback from the hematology and the laboratory departments was collected regularly to assess their satisfaction with the new system and identify any areas for improvement. Disagreement between the departments caused the cessation of the new system in week 14. Stakeholder feedback was collected to assess the problem and find solutions. The chief executive director of TASH, started to mitigate the issue as a third party by week 18 after a prior development and submission of an agreement document to both departments by week 16. Finally, on week 20, a thorough discussion was made between the two departments. The new system was initiated again by week 21, fully digitalized and working effectively five days a week.

**Emphasis on further encountered challenges:** Another challenge faced at the start of the speedy slide initiative was the poor quality of slides prepared by the laboratory. This led to dissatisfaction among hematology fellows and consultants, as it made the slides difficult to review. The reason was identified to be the poor quality of the Wright stain and a new quality Wright stain was proposed as a solution. In the meantime, improving the quality of the slides was discussed with the emergency department and was effective immediately. On the last week of week 24, the sixth week of PDSA 2, the hematology department fellow was changed, and the reviewed slides number decreased; this implicated that continuous advocacy of the system to newly changing personnel was important.

## Results

### Baseline

Before the initiation of the interventions, hematology patients in the emergency department had a median length of stay (LOS) of 144 hours. The median time to specialist consultation was 12 hours, and 53% of patients took incorrect turns while navigating to the X-ray room. Prior to the launch of the Speedy Slides initiative, the median turnaround time for peripheral smear results was 78 hours.

### PDSA cycle 1

**Fig 6:**
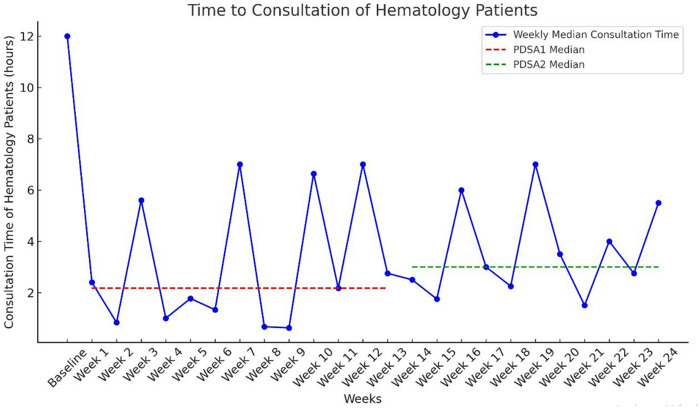
The weekly median time to consultation of hematology patients in the ED.

In the first cycle, two key interventions were introduced: floor markings and an online consultation platform. The online consultation platform significantly reduced the median time to consultation from 12 hours to 2.17 hours, an 81.9% decrease. Weekly median consultation times ranged from 0.625 to 7 hours, consistently remaining below the pre-intervention median, as demonstrated by the run chart (Fig 6). This reduction occurred despite the same number of staff and slightly higher hematology patient volumes compared to previous months. A shift was observed in the run chart, with six or more consecutive points below the pre-intervention median, revealing a non-random pattern consistent with a process shift.

**Fig 7:**
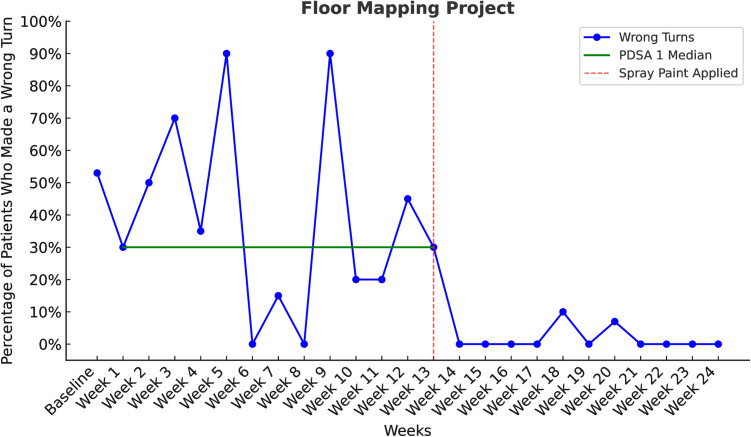
The average weekly percentage of patients who make a wrong turn on their way to the X-ray room.

The second intervention, floor markings, reduced the percentage of patients who took a wrong turn on their way to the X-ray room. The baseline wrong turn rate was 53%, and during PDSA Cycle 1, the median error rate decreased to 30%. Run chart analysis revealed a non-random pattern consistent with a process shift, with all post-intervention data points below the pre-intervention median (Fig 7).

**Fig 8:**
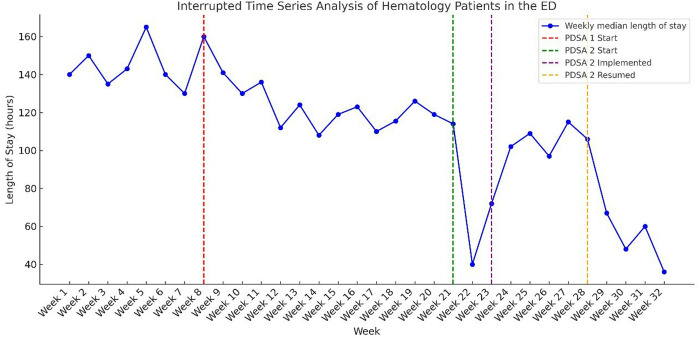
The weekly median length of stay of hematology patients in the ED.

Furthermore, the median LOS during PDSA Cycle 1 decreased from 144 hours to 119 hours, reflecting a 17.4% reduction. An Interrupted Time Series (ITS) analysis was conducted using Generalized Least Squares (GLS) regression, adjusting for autocorrelation with an AR(1) correlation structure. The model demonstrated a strong fit, explaining 94.5% of the variance (R² = 0.945). The Durbin-Watson statistic (2.10) confirmed that autocorrelation was well-controlled. ITS analysis indicated a significant immediate reduction in LOS (−18.4 hours, 95% CI: −27.9 to −8.9; p = 0.001) and a continued downward trend of −1.9 hours per week (95% CI: −3.2 to −0.6; p = 0.008), signifying sustained improvement over time (Fig 8).

### PDSA cycle 2

During PDSA Cycle 2, median consultation time slightly increased to 3.0 hours (range: 1.5 to 7 hours), though it remained well below the pre-intervention baseline of 12 hours. The run chart analysis showed that while consultation times remained lower than the baseline, a shift was not observed in PDSA Cycle 2 as compared to PDSA Cycle 1, indicating some process variability (Fig 6).

The “Speedy Slide” initiative resulted in a significant reduction in the median turnaround time for peripheral smears, from 78 hours to 23.25 hours, a 70% decrease. Additionally, the median LOS dropped drastically to 54 hours, representing a 62.5% decrease from the pre-intervention baseline and a 45% decrease from the first PDSA cycle median. The ITS analysis confirmed an immediate LOS reduction of −75.2 hours (95% CI: −88.1 to −62.3; p < 0.001) following PDSA 2 implementation. Even during the discontinuation period (Weeks 24–28), LOS remained stable (p = 0.933; 95% CI: −8.43 to 8.43), suggesting that some benefits of PDSA 2 were retained. When the initiative resumed (Weeks 29–32), LOS again decreased significantly (−49.34 hours, 95% CI: −62.67 to −36.01; p < 0.001), confirming its effectiveness and reproducibility (Fig 8).

Significant improvement in way finding errors was also observed in PDSA Cycle 2. From Week 14 onwards, following the application of spray paint, sustained reductions were recorded. Weeks 14–17 saw an immediate drop to 0%, with a minor increase to 10% in Week 18, followed by a return to near-zero levels in Weeks 19–24 (one fluctuation at Week 20 with 8%). A run chart analysis confirmed a clear process shift, with sustained reductions in way finding errors compared to the pre-intervention period (Fig 7).

Overall, both PDSA 1 and PDSA 2 effectively reduced LOS, but PDSA 2 had a much stronger and more immediate impact. The sustained reduction during the discontinuation period suggests meaningful system-level improvements. The AR(1) correlation structure successfully accounted for autocorrelation, ensuring the reliability of the findings. With an R² of 0.945, the ITS model explained 94.5% of the variation in LOS, indicating a strong fit and reliability of the results.

## Discussion

At Tikur Anbessa Specialized Hospital, a quality improvement initiative focused on shortening the time hematology patients spend in the Emergency Department (ED). This was pursued through three primary interventions: redesigning the lab system for peripheral smears, implementing an online consultation platform, and developing a floor map for patient navigation.

The “Speedy Slides” project dramatically reduced the time needed to obtain peripheral smear results, bringing it down from 78 hours to just over 23 hours. This change enabled quicker diagnoses and treatments, which contributed to a shorter stay for hematology patients in the emergency department. The Interrupted Time Series (ITS) analysis further confirmed the robustness of this intervention, with an R² of 0.945, indicating that 94.5% of the variation in length of stay (LOS) was explained by the model—a finding that underscores the systemic impact of streamlined lab processes. Shen and Lee also observed a similar impact when they enhanced the lab system at Singapore General Hospital, reducing triage wait times from 18 minutes to 13 minutes [7]. Both studies highlight the critical role of efficient lab processes in enhancing patient navigation and reducing LOS in emergency settings, emphasizing that timely diagnostics are foundational to effective patient management. Notably, the stability of LOS during the 5-week discontinuation of PDSA 2 suggests that workflow adaptations, once embedded, can yield sustained efficiencies even during implementation pauses.

The new consultation platform reduced the median time to consultation from 12 hours to 2.17 hours, demonstrating an 81.9% improvement. This intervention enhanced the efficiency of patient care by enabling faster communication with senior specialists. Run chart analysis confirmed a sustained shift in consultation times, with all post-intervention data points remaining below the pre-intervention median, reflecting a durable process change. This finding is consistent with other studies that have explored the use of online consultation platforms to streamline communication between departments and patient navigation in emergency settings. A study by Ravi et al. integrated a messaging system into the electronic health record (HER) order entry process, such that placing a consult order simultaneously paged the consultant [21]. They found that this intervention was associated with a 15-minute reduction in median time to consult initiation and a 52-minute reduction in median ED length of stay (LOS). Similarly, our study showed the potential of a redesigned online consultation platform to reduce consultation times and improve patient outcomes with further refinements. However, qualitative reports of temporary staff resistance during PDSA 2 implementation highlight the need to balance efficiency gains with workforce well-being in future iterations.

The implementation of floor markings reduced patient confusion and improved navigation within the ED. This intervention decreased the percentage of patients taking wrong turns, contributing to smoother patient navigation and reduced LOS. Post-intervention data revealed a sustained reduction to near-zero levels (Week 14–24), with a minor fluctuation to 10% and 8% in week 18 and 20 respectively, and a run chart confirming a statistically significant shift—a finding that underscores the long-term impact of environmental design on patient navigation. Similar navigation aids have been implemented in other EDs, where clear signage and floor maps have been shown to reduce patient misdirection and improve throughput. For instance, a study highlighted that directional guides in the form of floor markings facilitate easier navigation for patients and visitors, leading to a more streamlined patient experience and potentially shorter LOS due to reduced time spent searching for departments or services [22]. While our study focused on operational metrics, future work should explore clinical outcomes (e.g., mortality, readmissions) to holistically assess the health implications of reduced LOS.

### Limitations

This study has several limitations. First, while baseline length of stay (LOS) used an eight-week retrospective dataset, secondary measures (consultation time, smear turnaround, navigation efficiency) relied on a one-week observational study that may not capture variability, despite aligned staffing/volume. Second, Hawthorne effects likely amplified improvements due to staff awareness. Third, though patient satisfaction improved, we did not formally quantify increased workloads (e.g., hematology fellows, lab technicians) despite qualitative reports of strain.

Fourth, the “Speedy Slide” initiative was paused for five weeks due to unsustainable lab workloads, with data excluded during this period, and ultimately discontinued post-study due to unresolved interdepartmental tensions. This limits insights into sustainability during inactive phases. Fifth, the 24-week study period precludes long-term conclusions, and the lack of a control group (e.g., standard care comparison) obscures isolation from external factors like seasonal demand. Finally, clinical outcomes (mortality, diagnostic accuracy) were unassessed, leaving health implications unclear.

Future quality improvement studies should prioritize multi-center, long-term studies to assess scalability across settings, integrate clinical outcomes (e.g., mortality) and staff workload metrics, and leverage cost-effectiveness analyses/AI tools (e.g., lab workflow analytics) to optimize resources. Structured implementation with conflict-resolution protocols would ensure equitable, staff-sensitive adoption in low-resource environments.

## Conclusion

In conclusion, the quality improvement project at Tikur Anbessa Specialized Hospital demonstrates that targeted interventions can lead to significant enhancements in patient care and operational efficiency. By incorporating comparative analyses from similar studies, this discussion underscores the importance of efficient processes, effective communication, and stakeholder engagement in achieving sustained improvements in healthcare delivery. However, the initiative did not achieve sustained improvement in ED LOS. Data gaps during a five-week pause and the discontinuation of the “Speedy Slide” due to interdepartmental issues highlight sustainability challenges. Future efforts should ensure continuous monitoring, stakeholder engagement, and staff well-being.

## Supporting information

S1 File Weekly median consultation time data. (XLSX)

S2 File Weekly median length of stay data. (XLSX)

S3 File Weekly median turnaround time data. (XLSX)

S4 File Wrong turn percentage data. (XLSX)
